# The Trust Game for Couples (TGC): A new standardized paradigm to assess trust in romantic relationships

**DOI:** 10.1371/journal.pone.0230776

**Published:** 2020-03-26

**Authors:** Tobias Kleinert, Bastian Schiller, Urs Fischbacher, Laura-Anne Grigutsch, Nicolas Koranyi, Klaus Rothermund, Markus Heinrichs

**Affiliations:** 1 Laboratory for Biological and Personality Psychology, Department of Psychology, University of Freiburg, Freiburg, Baden-Wuerttemberg, Germany; 2 Freiburg Brain Imaging Center, University Medical Center, University of Freiburg, Freiburg, Baden-Wuerttemberg, Germany; 3 Department of Economics, University of Konstanz, Konstanz, Baden-Wuerttemberg, Germany; 4 Thurgau Institute of Economics, Kreuzlingen, Thurgau, Switzerland; 5 Department of Psychology, Friedrich-Schiller-University of Jena, Jena, Thuringia, Germany; University of East Anglia, UNITED KINGDOM

## Abstract

Trust between couples is a prerequisite for stable and satisfactory romantic relationships. However, there has been no valid research tool to assess partner-specific trust behavior including costly investments in the trustworthiness of the romantic partner. We here present a comprehensive validation of the newly developed Trust Game for Couples (TGC) by means of various self-report and implicit relationship-related measures. The TGC operationalizes trust by measuring an individual’s willingness to invest his or her own financial resources in pro-relationship attitudes of their romantic partner (collected by dichotomous responses to relationship-relevant items, e.g., answering yes to “I am absolutely sure that I love my partner”). Thirty-five healthy couples between 20 and 34 years completed the TGC in an interactive (both partners present), but anonymous setting (no information on the partner’s responses revealed). Trust, as measured by the TGC, correlates positively with self-reported trust, satisfaction, and felt closeness in the relationship, but not with general interpersonal trust, confirming both its convergent and discriminant validity. In addition to explicit criteria for construct validity, implicit measures of partner valence and confidence explained variance in the TGC, demonstrating that it constitutes an economical measure of implicit and explicit ingredients of trust between couples. In sum, the TGC provides a novel, specific behavioral tool for a sensitive assessment of trust in dyadic relationships with potential for numerous research fields.

## Introduction

According to major psychological theories, including attachment theory [1] and the dyadic model of trust [2], trust is one of the most important qualities for developing and maintaining long-term romantic relationships (for reviews, see [3,4]). In attachment theory, trust is developed as a trait-like internal representation in reaction to early experiences with caregivers determining attachment styles in later (romantic) relationships [1], while in the dyadic model of trust, it represents a person-situation interaction with an interdependent romantic partner combining elements of a trait and state [2]. Empirical research has confirmed the importance of trust in romantic relationships by demonstrating that high levels of self-reported trust in the romantic partner relate positively to love and happiness [5], a positive perception of relationship quality and daily interactions [6], pro-relationship acts [6,7], and the feeling of commitment towards the relationship [7]. In addition, associations between trust and the evolutionarily well-conserved neuropeptide oxytocin highlight the vital importance of trust for the survival of the human species from an evolutionary perspective [8]. Despite the complexity of the trust construct [4], different interpretations share several key elements summarized in the following working definition: First, trust involves confidence, e.g., that personal needs will be fulfilled [2,9,10]. Second, it requires an investment of personal resources (e.g., emotions, time, money) with the anticipation of future advantages, creating interpersonal dependency towards a trustee [8,11,12]. Third, trust involves a risk, as the trustee might choose to disregard expectations or abuse investments [5,13,14]. Despite providing highly valuable information, existing self-report measures of partner-specific trust lack these key characteristics of trust behavior in actual social interactions between romantic partners. Further, they regard the construct as a purely subjective belief or as an attitude that is only introspectively accessible, which might be confounded by socially desirable response tendencies [15,16]. In the current study, we present the Trust Game for Couples (TGC) as a new interactive tool providing an ecologically valid measure of partner-specific trust behavior involving actual decisions with real monetary consequences.

An ecologically valid measure of trust involving costly behavior already exists in the domain of measuring trust between strangers. In the well-known “classical” trust game, the player in the role of investor can transfer a share of an initial monetary endowment to an anonymous partner, the trustee ([8,11]; for recent research on trust behavior also see [17,18]). If the investor transfers money, the total amount available to be shared between the two players increases. Then, the trustee is informed about the investor’s transfer and can either choose to honor the investor’s trust by sharing the monetary profit or keep all the money for himself. The initial monetary investment handed over by the investor to the trustee without knowing about his or her potential back-transfer represents a costly investment in the trustworthiness of the interaction partner, which is considered trust behavior. Importantly, to prevent reciprocal or reputational considerations from confounding the investor’s trust decision, players interact only once, and interaction partners remain anonymous. These constraints illustrate the difficulty of applying the trust game to assess trust in interactions between romantic partners, as it is simply impossible to create an anonymous setting with a single interaction sequence between people who know each other well and who know that they will continue to interact with each other after the assessment. Nobody is likely to risk a couple conflict by failing to transfer the full endowment.

To address this issue, we developed the TGC as a new measure of trust between romantic partners involving costly behavior. The main idea of this game paradigm is that trusting partners should be willing to invest their own (financial) resources in pro-relationship attitudes of their romantic partner. These attitudes are collected by dichotomous responses to relationship-relevant items. For example, high trust is indicated by investing a large amount of resources in the partner responding “yes” to “I am absolutely sure that I would never cheat on my partner”, while low trust is indicated by investing resources in the partner responding “no”. To increase the likelihood of “true” responses to these relationship-related items, the TGC is introduced to participants as a partner decision game, in which a joint profit can be maximized by correctly estimating the partner’s responses to relationship-relevant items. Both partners are advised to answer honestly to enable accurate estimations by their partner and thus increase their joint profit. Critically, at no point during the experiment are partners allowed to communicate, and to guarantee anonymity and thus minimize tendencies towards socially desirable answers, they get no feedback about their partner’s responses.

**Fig 1:**
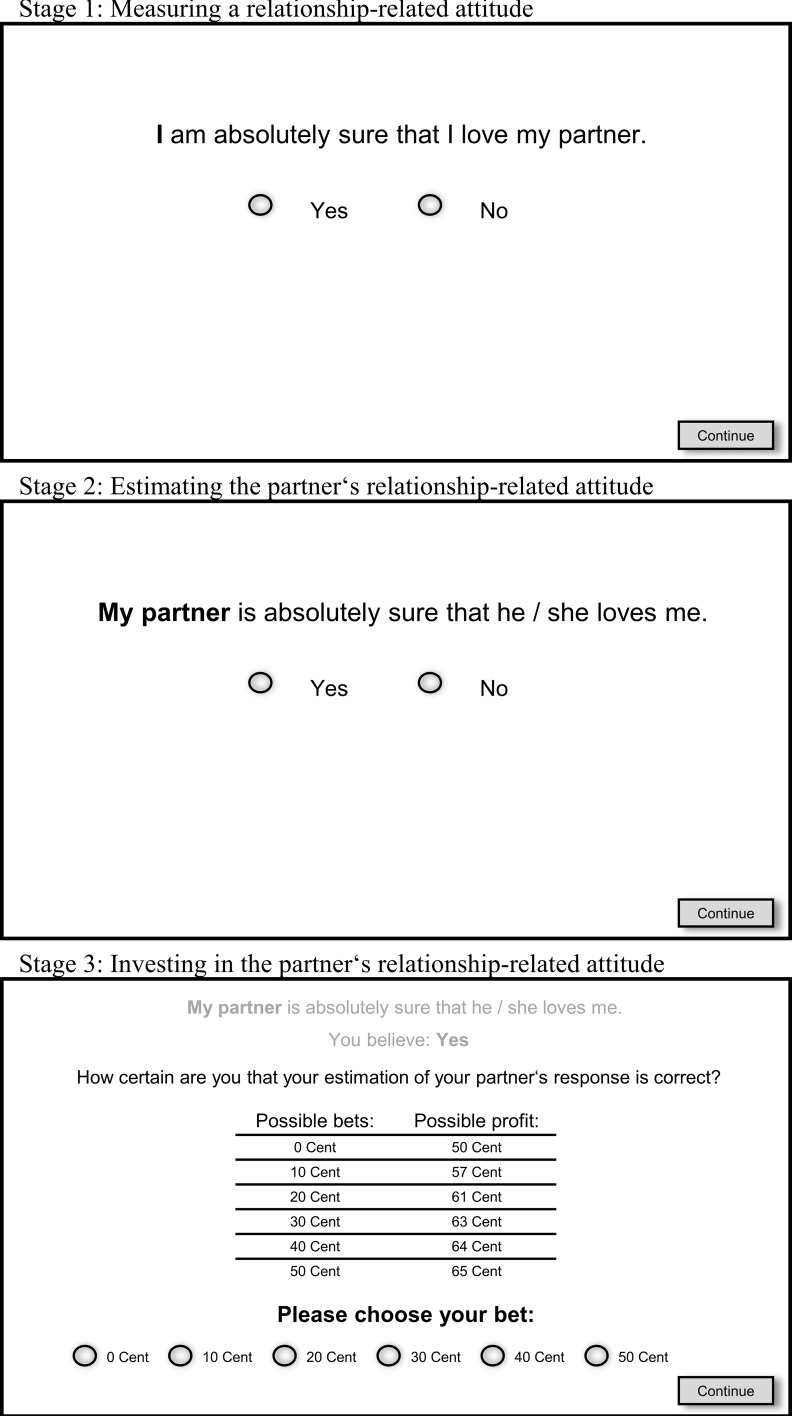
Example round of the TGC. One round consists of three stages. *Stage 1*: *Measuring a relationship-related attitude*. Each partner has to respond with “Yes” or “No” to a relationship-related statement. For example, partners have to decide whether they agree with the statement “I am absolutely sure that I love my partner” which would represent the pro-relationship attitude from the perspective of the partner. *Stage 2*: *Estimating the partner’s relationship-related attitude*. Each partner has to estimate whether his or her partner agreed or disagreed with the previous relationship-relevant statement. For example, partners have to decide whether they think that their partner agreed with the statement “I am absolutely sure that I love my partner”, which would mean that they believe in their partner’s pro-relationship attitude. *Stage 3*: *Investing in the partner’s relationship-related attitude*. Both partners can invest their own resources in correctly estimating their partner’s response (presented as a reminder in gray at the top of the screen) in order to increase the shared profit. Each partner has an initial endowment of 50 cents. Both partners can choose to invest 0, 10, 20, 30, 40 or 50 cents in order to increase their profit from 50 to a maximum of 65 cents. From these investments, a trust score is calculated that rises in case of larger investments in the partner’s pro-relationship attitudes and decreases in case of larger investments in the partner’s anti-relationship attitudes (for details, see Materials and methods and Fig 2).

Each round of the TGC consists of three stages played simultaneously, creating an immediate couple interaction (see Fig 1). First, both partners respond to a relationship-relevant item (see Fig 1, stage 1). Second, both partners estimate their partner’s response (see Fig 1, stage 2). Third, both partners can invest their own resources to bet on the correct estimation of their partner’s response in order to increase their joint profit (see Fig 1, stage 3). Thus, a couple’s profit depends on the match between the estimated and actual responses by partners, while trust in the partner is operationalized without the knowledge of participants by means of an individual *trust score*, which rises in case resources are invested in pro-relationship attitudes of the partner and decreases in case resources are invested in anti-relationship ones. For example, investing resources in the partner’s answering “yes” to the statement “I am absolutely sure that I would never cheat on my partner” would result in points added to one’s own trust score, while investing in the partner’s answering “no” would result in points subtracted from one’s own trust score. The number of points added to or subtracted from the trust score depends on the amount of resources invested, representing how certain one is in the partner’s pro- or anti-relationship attitudes (for details, see Method).

We hypothesized that the TGC would demonstrate satisfactory reliability as indicated by the internal consistency of its distinct items. Second, we hypothesized that the trust scores of romantic partners would correlate and that the mean trust score would be positive, assuming considerable levels of interdependent trust in intact relationships lasting at least one year in our sample [5]. Third, we hypothesized that the TGC would possess high discriminant validity as indicated by non-significant associations with general interpersonal trust ([19]; we measured interpersonal trust using the German Kurzskala Interpersonales Vertrauen, for the English version see Appendix C in [20]) and with socially desirable response tendencies (Balanced Inventory of Desirable Responding; [16], German version by [21]). Fourth, we hypothesized that the TGC would possess high convergent validity as indicated by significant positive associations with several relationship-related self-report measures, such as trust in the relationship (Dyadic Trust Scale; [22]; German version Vertrauen in der Partnerschaft by [23]), satisfaction with the partner (Partnerschaftsfragebogen [24]), satisfaction with the partnership (Relationship Assessment Scale [25], German version by [26]) and felt closeness with the partner (Inclusion of Other in the Self scale [27]). Fifth, we hypothesized that the TGC would expand upon information provided by self-report measures, as indicated by incremental variance explained in the TGC by implicit relationship-related measures such as partner valence (Partner Implicit Association Test; see Materials and methods) and confidence in knowing the partner (operationalized by response times while estimating partner responses in the TGC; see Materials and methods). Thus, we tested whether the TGC constitutes a specific and economical measure of both explicit and implicit ingredients of trust behavior between romantic partners.

## Materials and methods

### Sample

A total sample of 35 heterosexual couples aged 20 to 34 years participated in this study, having been in a romantic relationship for at least one year. Couples with children and pregnant women were not allowed to participate to avoid heterogeneous endocrine preconditions having a potential influence on trust-decisions [28]. Further exclusion criteria were insufficient fluency in the German language, current or previous history of neurological and psychiatric disorders, and alcohol, nicotine, or drug abuse. We excluded five of the initial 70 participants for data analyses because of language or comprehension problems during the experiment (*n* = 3), or explicit reports of strategic gameplay in the TGC (*n* = 2). This resulted in a final sample size of *N* = 65 participants (33 female; note that partners of excluded participants were not automatically excluded, as all measures were calculated individually) with an average age of *M* = 23.35 years (*SD* = 3.14, *Range* = 20–34). A post hoc power analysis (for HLM using Monte-Carlo simulations with 1000 repetitions; [29]) based on the significant associations found in this study revealed a statistical power ranging from 61.70% (association of our trust score with Closeness to Partner; IOS) to 99.30% (association of our trust score with Togetherness / Communication; PFB TC). Couples had been together for 2.62 years on average (*SD* = 1.24, *Range* = 1-6.25 years), and reported high partnership satisfaction in the Relationship Assessment Scale (RAS; [25]; *M* = 4.40, *SD* = .42, *Range* = 3.29-5.00). The Ethics Committee of the University of Freiburg approved this study, which was conducted according to the principles expressed in the Declaration of Helsinki. All procedures were carried out with the adequate understanding and informed written consent of the participants.

### Procedure

We recruited participants via flyers, bulletins, and in online social-media platforms. Both partners completed an online screening questionnaire to assess demographic data and exclusion criteria. Eligible couples were contacted by phone applying a standardized guideline to provide further information on the study and arrange an appointment for the experimental session.

Experimental sessions took 90 minutes and were conducted in a group laboratory specifically designed for social interaction experiments. Two trained study assistants ran the experiment with one couple per session. Upon arrival, partners received information on the experimental procedure and instructions not to talk or otherwise interact with each other during the experiment (duration: 10 min.). Couples sat at opposite computers, separated by partitions and wearing sound-dampening headphones. Next, they received detailed instructions on the TGC and answered control questions testing their understanding of the game principles (see S1 Fig, 10 min.). Then, couples executed the TGC (20 min.), followed by a Partner Implicit Association Test (P-IAT, 20 min.). Finally, both partners completed a battery of questionnaires (30 min.). After the experiment, couples received their shared payment (40 € basic payment plus monetary reward of the TGC) of 56.38 € on average (*SD* = .940, *Range* = 54.09–58.21).

### The Trust Game for Couples (TGC)

The TGC was programmed with z-Tree [30] and is freely available from the corresponding author on request in English and German. The TGC consists of 15 rounds; nine thereof include relationship-relevant items and six include distractor-items that were integrated to conceal the paradigm’s true purpose (see S1 Table for the full item set). Each round of the TGC consists of three stages: First, both partners respond to a relationship-relevant item (see Fig 1, stage 1). Second, both estimate their partner’s response (see Fig 1, stage 2). Third, both partners can invest their own resources to bet on having correctly estimated their partner’s response in order to increase the shared profit (see Fig 1, stage 3; and S2 Fig for detailed information on participants’ investments). To prevent pressure on slower players through their partner’s faster decision-making, random waiting-stage screens (“Please wait until the experiment continues”) were presented for 1–4 seconds after each stage for both players, thus concealing who had completed that stage first. The instructions (see S1 Fig) specifically emphasized that all responses during the TGC would remain anonymous and be visible to neither their own partner nor the study assistants. Participants were further informed that the individual profits of both partners would be combined to enable a joint profit per couple, which meant that one partner could not derive any information about their partner’s individual responses or profits. Participants were advised to respond spontaneously and honestly because this would maximize the probability that their partner correctly estimates their response and thus maximizes the joint profit. Finally, they received detailed information on the payoff rules and the structure of each playing round.

**Fig 2:**
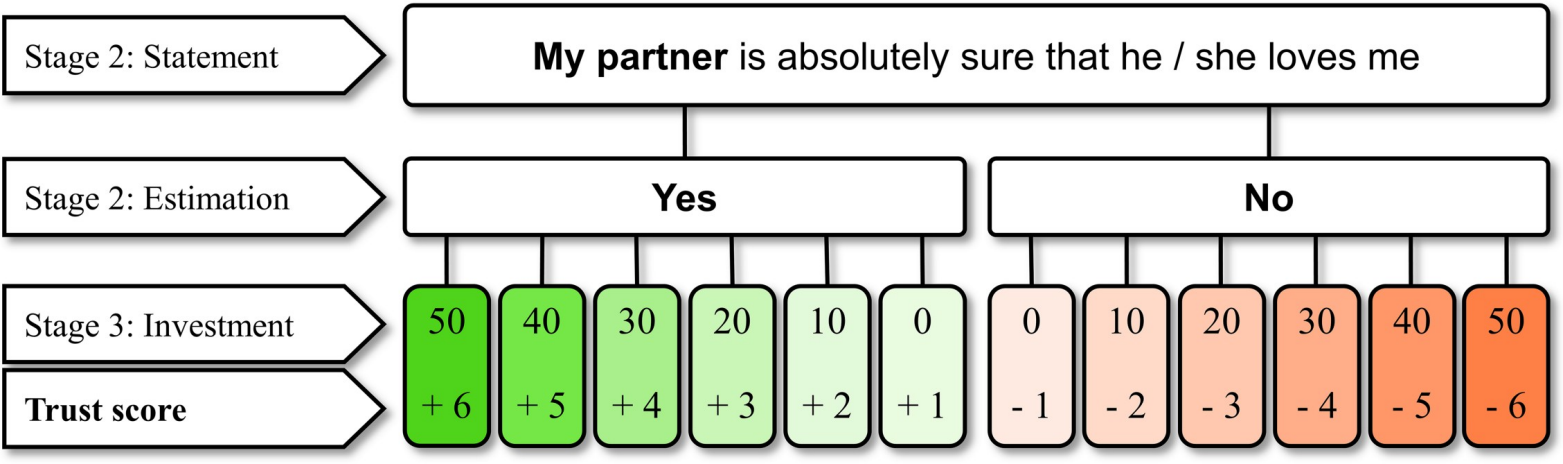
Calculation of the trust score in the TGC. After having estimated their partner’s response to the relationship-relevant item (stage 2), each partner can choose to invest 0 to 50 cents in this estimation to be true (stage 3). In each round, a separate trust score is calculated based on the estimation of the partner’s response and the investment. Investing in a pro-relationship attitude of the partner results in a positive score (+1 for investing 0 cents, to +6 for investing 50 cents), while investing in an anti-relationship attitude of the partner results in a negative score (-1 for investing 0 cents, to -6 for investing 50 cents). The total trust score is calculated as the sum of the nine separate trust scores from each round in response to the nine relationship-relevant items, ranging between -54 and +54 points. Note that this calculation is independent of whether the estimations of the partners’ responses were correct or incorrect, which is only relevant for profit calculation (see Table 1).

To operationalize trust in the romantic partner in the TGC, a *trust score* is calculated, which represents the participant’s willingness to invest money in their partner’s pro-relationship attitudes collected via dichotomous responses to several relationship-relevant items. In each round (i.e., for each item), a *separate trust score* is calculated based on the estimation of the partner’s response in stage 2 and the investment in stage 3. The estimation of the partner’s response defines the polarity, and investments the absolute value of the separate trust score for each item. Investing in the partner’s pro-relationship attitudes results in a positive separate trust score (+1 for investing 0 cents to +6 for investing 50 cents), while investing in the partner’s anti-relationship attitudes results in a negative separate trust score (-1 for investing 0 cents to -6 for investing 50 cents; see Fig 2). The total trust score is calculated as the sum of the nine separate trust scores from each round in response to the nine relationship-relevant items, ranging between -54 and +54 points. Note that this calculation does not consider whether estimations of the partners’ responses were correct or not, which is why the trust score does not necessarily reflect how well one knows his or her partner (being relevant only for calculating the shared profit). Rather, a high trust score reflects strong confidence in the partner’s pro-relationship attitudes resulting from costly investments in the partner’s pro-relationship responses to relationship-relevant situations involving the anticipation of future (monetary) profit, and the risk that expectations might go unfulfilled (an operationalization that is in line with the working definition of trust provided in our introduction). On the other hand, a low positive trust score reflects a lack of confidence in the partner’s pro-relationship attitudes, while a negative trust score reflects confidence in the partner’s anti-relationship attitudes (possibly originating in trust violated by the partner earlier in the relationship).

A couple’s joint profit depends on the match of responses by both partners (i.e., comparing the own estimation of the partner’s response in stage 2 with his or her actual response in stage 1), and is calculated by combining both partners’ individual profits from all items. Per round, each partner receives the amount of resources kept (50 cents minus investment) plus the profit from investments made from correctly estimating their partner’s response (ranging from 17 cents for investing 10 cents to 65 cents for investing 50 cents). Note that higher investments are more risky because the marginal winning probability is decreasing. This also helps avoiding ceiling effects (for details, see Table 1).

**Table 1:** Calculation of profits in the TGC.

**Bet**	0	10	20	30	40	50
**Losing Profit**	50	40	30	20	10	0
**Winning Profit**	50	57	61	63	64	65

Winning profits result from a correct estimation of the partner’s response during stage 2, losing profits from an incorrect one. Note that profits are calculated independently for each partner, but both partners only receive information about their joint profit after the experiment in order to guarantee the anonymity of responses during the TGC.

### Discriminant validation criteria

The short-scale KUSIV3 (Kurzskala interpersonales Vertrauen; [20]; *α* = .85) assesses general interpersonal trust in others; it consists of 3 items with 5-point Likert scales. The Balanced Inventory of Desirable Responding (BIDR; [16]) assesses two dimensions of desirable responding; Self-Deceptive Enhancement (SDE) and Impression Management (IM). Participants completed the German version containing 20 items with 7-point Likert scales, with 10 items assigned to each of the two subscales ([21]; *α* = .62 (SDE), *α* = .65 (IM)).

### Convergent validation criteria

#### Relationship-related self-report measures

The Dyadic Trust Scale [22] was developed as a unidimensional questionnaire for assessing trust in a partnership. Our participants completed the German version, the VIP (Vertrauen in der Partnerschaft; [23]; *α* = .93), consisting of 8 items with 4-point Likert scales. The RAS (Relationship Assessment Scale; [25]) is a seven-item questionnaire for the unidimensional measurement of general satisfaction with the partnership using 5-point Likert scales. Participants completed the German version of the questionnaire ([26]; *α* = .81). The PFB (Partnerschaftsfragebogen; [24,31]; *α* = .93) assesses acute satisfaction with the partner’s behavior on three dimensions: *Quarreling* (QU; *α* = .88), *Tenderness* (TD; *α* = .91) and *Togetherness/Communication* (TC; *α* = .85), with 10 items per subscale. The questionnaire includes 30 items, answered by 4-point Likert scales addressing the frequency of a given behavior. The subscales TD and TC are positive indicators for partner satisfaction, while QU is a negative indicator. Finally, we included felt closeness with the partner, which was operationalized by the IOS (Inclusion of Other in the Self scale; [27]; *rtt* = .85). The IOS is an abstract single-item pictorial scale in which participants choose one out of 7 pictures, each showing two increasingly overlapping circles that represent the self and the partner.

#### Implicit relationship-related measures

The *Partner Implicit Association Test* (P-IAT) is an adaptation of the classic Implicit Association Test with a 7-block structure [32]. It indirectly assesses partner valence in contrast to an attractive alternative by comparing the strength of associations of target categories (Partner vs. Alternative) with attribute categories (Positive vs. Negative) using response times. In the P-IAT, participants have to correctly and quickly classify stimuli belonging to the four distinct categories with two response-keys. Attribute stimuli were pictures with positive and negative valence (taken from the International Affective Picture System; [33]), target stimuli were words that were associated with their own partner or an attractive alternative. An individual set of stimuli was used for the category Partner, including the first and last name, a characteristic hobby, and a characteristic trait of the partner (e.g., Max, Mustermann, Snowboard, Humorous). Analogous information was provided on an attractive alternative whose identity was individually selected from four pictures of the opposite sex (attractive faces taken from the Chicago Face Database; [34]). In the first two blocks, participants practiced the correct classification of positive vs. negative pictures (16 trials) and partner- vs. alternative-related word stimuli (16 trials). In the third and fourth, *congruent* blocks (32 and 64 trials), stimuli of the categories Positive and Partner were assigned to one response key, and stimuli of the categories Negative and Alternative to another. In the fifth block, a reversed key-assignment was practiced for the categories Partner and Alternative (16 trials). In the sixth and seventh, *incongruent* blocks (32 and 64 trials), stimuli of the categories Positive and Alternative now shared one response key, while stimuli of the categories Negative and Partner shared the other. The difference between response times in congruent and incongruent blocks represents the strength of the implicit association between the categories Partner and Positive (and between Alternative and Negative) compared to the reversed associations. In practice blocks (blocks 1, 2, 3, 5, and 6), categories were presented on top of the screen for assistance, and response times > 1000ms were followed by the feedback “Too slow!” in red letters to promote quick answers. If participants answered incorrectly, a red “X” was shown until the correct response was forthcoming. Final D scores were calculated according to the improved scoring algorithm [35]. Please refer to the S2 Table, S3 Fig, S4 Fig and S5 Fig for detailed information on the Partner Implicit Association Test.

Second, we included *implicit confidence* (IC) in knowing the partner, which was operationalized by mean individual response times to trust-relevant items in the TGC during stage 2. For an easier interpretation of our IC measure, we used polarity reversed response times, with larger values of the IC variable representing shorter response times, i.e., higher confidence in knowing the partner. It is plausible that higher confidence in estimating the partner’s response correctly leads to faster response times, as easy choices should take less cognitive effort [36,37].

#### Statistical analyses

All analyses were conducted with the software packages SPSS (23rd ed.) and RStudio (R version 3.5.0, RStudio version 1.1.453). First, we calculated individual trust scores for each participant. Next, we tested the internal consistency of trust decisions in the TGC by calculating Cronbach’s Alpha using the nine separate trust scores. As a further check for the trust score’s validity, we calculated the intra-class-correlation (ICC) of trust scores between partners, anticipating that a considerable amount of variance would be explained by couple-membership. Next, we used hierarchical linear modeling (HLM; [38]), with random variation of intercepts between couples to test for significant predictors of the trust score and thus test the TGC’s validity. Prior to our analyses, metric variables were z-standardized to obtain interpretable model-coefficients for HLM. Following the recommended procedure by Nakagawa and Schielzeth [39], we calculated marginal R-squared values (R^2^_m_), representing the estimated amount of variance explained by fixed factors [40]. To test the TGC’s validity, we ran two main analyses: In analysis 1, we included relationship-related self-report measures in single-predictor models of the trust score. With regard to discriminant validity, we expected non-significant predictions of the trust score by general interpersonal trust (KUSIV3), impression management (BIDR-IM), and self-deceptive enhancement (BIDR-SDE). With regard to convergent validity, we expected significant predictions of the trust score by trust in the partnership (VIP), satisfaction with the partnership (RAS), satisfaction with the partner (PFB) and closeness with the partner (IOS). Finally, we included all self-report measures as predictors in one model in order to test their combined predictive power (Explicit Measures Model). In analysis 2, we added step-by-step our two implicit measures (partner valence, confidence in knowing the partner) as predictors to the Explicit Measures Model in order to test whether these implicit measures are capable of explaining incremental variance in the TGC trust score. Thus, we tested for the combined predictive power of all explicit and implicit relationship-related measures in our study for predicting the TGC trust score.

## Results

### Reliability

To assess the TGC’s reliability, we calculated the trust score’s internal consistency by applying the nine separate trust scores of relationship-relevant items. The resulting Cronbach’s Alpha amounts to *α* = .746, indicating the TGC trust score’s reliability.

### Trust score properties

**Fig 3:**
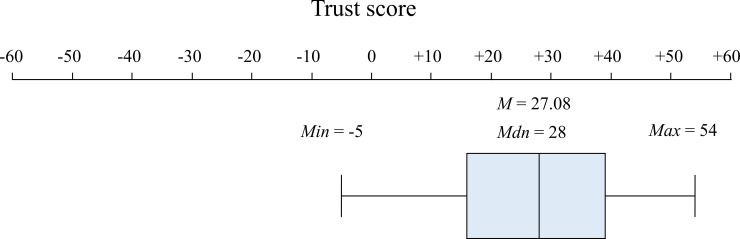
Distribution of the trust score in a box-plot. *N* = 65. Possible range of the trust score: -54 to +54. Note that the trust score exhibits considerable variation and its mean score is clearly positive. See S2 Fig for detailed information on investments, which we used to calculate the trust score.

As initial checks of the trust score’s validity, we analyzed the descriptive statistics of the trust score in our sample and the correlation of trust scores between romantic partners. As expected, the mean trust score was positive, and revealed considerable variation as indicated by its normal distribution (*M* = 27.08, *SD* = 15.79; ANOVA testing the trust score’s statistical difference from zero: *F(1*,*64)* = 191.16, *p* < .001, *η*^*2*^ = .749; Shapiro-Wilk test for normal distribution: *p* = .198), ranging from -5 to the maximum possible score of 54 points (Fig 3; see S2 Fig for descriptive properties and distributions of separate trust scores for all nine relationship-relevant items). Finally, we detected an intra-class correlation (ICC) between trust scores of romantic partners of *ICC* = .443 indicating that a substantial portion of variance in trust scores is due to systematic differences in average trust levels between couples. This finding already attests to the specificity of partnership-related trust as compared to general interpersonal trust.

### Discriminant validity of the trust score

Testing for the TGC trust score’s discriminant validity, we analyzed its prediction by self-report measures of general interpersonal trust (KUSIV 3) and socially desirable response tendencies (BIDR subscales Impression Management and Self-Deceptive Enhancement). In line with our hypothesis, we detected no significant relationships (all *p* > 0.072; for details see Table 2), demonstrating the discriminant validity of the trust score.

**Table 2:** Associations between the TGC trust score and explicit and implicit relationship-related measures in single predictor models.

**Explicit relationship-related measures**						
**Predictor**	**Construct**	**b**	**SDE**	**t(30)**	**p**	**R**^**2**^_**m**_
KUSIV3	Interpersonal Trust	.061	.121	.503	.619	.004
BIDR-IM	Impression Management	-.077	.111	-.696	.492	.006
BIDR-SDE	Self-Deceptive Enhancement	.214	.114	1.87	.072	.047
VIP	Partnership Trust	.337	.118	2.85	**.008**	**.121**
RAS	Partnership Satisfaction	.306	.124	2.47	**.020**	**.111**
IOS	Closeness with Partner	.274	.120	2.29	**.029**	**.077**
PFB	Partner Satisfaction	.327	.122	2.68	**.012**	**.111**
PFB TC	Togetherness / Communication	.500	.111	4.49	**< .001**	**.257**
PFB TD	Tenderness	.174	.119	1.46	.154	.031
PFB QU	Quarreling	-.057	.134	-.426	.674	.003
**Implicit relationship-related measures**						
P-IAT	Implicit partner valence	.188	.114	1.64	.112	.037
IC	Implicit confidence	.291	.110	2.64	**.013**	**.092**

b = regression coefficients, SDE = standard errors, t = t-values with degrees of freedom, p = p-values, R^2^_m_ = marginal R-squared values. Results of single predictor hierarchical linear models with explicit and implicit relationship-related measures predicting the TGC trust score. Bold values indicate significant associations with the TGC trust score.

### Convergent validity of the trust score

We analyzed the TGC trust score’s convergent validity by measuring how accurately it predicted several relationship-related self-report measures in separate single predictor models. In line with our hypotheses, trust in the partnership (VIP; *b* = .337, *t*(30) = 2.85, *p* = .008), satisfaction with the partnership (RAS; *b* = .306, *t*(30) = 2.47, *p* = .020), felt closeness with the partner (IOS; *b* = .274, *t*(30) = 2.29, *p* = .029) and satisfaction with the partner (PFB; *b* = .327, *t*(30) = 2.68, *p* = .012) were significant positive predictors of the TGC trust score. The latter effect was primarily driven by the PFB subscale Togetherness and Communication (*b* = .500, *t*(30) = 4.49, *p* < .001), whereas the subscales Quarreling and Tenderness did not predict the trust score significantly (both *p* > .154, see Table 2). Notably, all self-report measures were included as predictors in one Explicit Measures Model (see Explicit Measures Model in Table 3), which demonstrates the self-report measures’ high multicollinearity (only the IOS was a significant predictor in this model; *b* = .282) and explains 28% of variance in the trust score. Taken together, the significant positive associations with all the relationship-related measures we used provide strong evidence of the trust score’s convergent validity by demonstrating that participants with higher trust in their partner (as determined by the TGC) also report higher trust in the partnership, higher satisfaction with the partnership, higher satisfaction with their partner, and a higher felt closeness with their partner. Meanwhile, large portions of the variance in the trust score remained unexplained, suggesting that the TGC might capture additional ingredients of trust beyond those that classic self-report measures capture, such as implicit perceptions of the partner and partnership.

**Table 3:** Multiple predictor models of the TGC trust score.

		Explicit Measures Model					Explicit Measures + P-IAT					Explicit Measures + P-IAT + IC				
Predictor	Construct	b	SDE	t(24)	p	R^2^_m_	b	SDE	t(23)	p	R^2^_m_	b	SDE	t(22)	p	R^2^_m_
KUSIV3	Interpersonal trust	.073	.122	.598	.555	**.277**	.113	.118	.962	.346	**.370**	.087	.109	.767	.434	
BIDR-IM	Impression Management	-.193	.115	-1.67	.108		-.175	.112	-1.56	.131		-.115	.105	-1.09	.286	
BIDR-SDE	Self-Deceptive Enhancement	.223	.115	1.95	.064		**.247**	**.110**	**2.24**	**.035**		.196	.104	1.89	.073	
VIP	Partnership Trust	.170	.146	1.17	.255		.241	.141	1.70	.102		.164	.132	1.25	.226	
RAS	Partnership Satisfaction	.030	.145	.208	.837		-.047	.141	-.336	.740		-.036	.128	-.281	.782	
PFB	Partner Satisfaction	.158	.156	1.01	.322		.168	.148	1.13	.268		.217	.137	1.59	.127	
IOS	Closeness with Partner	**.282**	**.120**	**2.34**	**.028**		**.296**	**.115**	**2.57**	**.017**		**.329**	**.106**	**3.10**	**.005**	
P-IAT	Implicit Partner Valence	-	-	-	-		**.285**	**.109**	**2.61**	**.016**		**.346**	**.102**	**3.39**	**.003**	
IC	Implicit Partner-Confidence	-	-	-	-		-	-	-	-		.342	.101	**3.37**	**.003**	**.490**

b = regression coefficients, SDE = standard errors, t = t-values with degrees of freedom, p = p-values, R^2^_m_ = marginal R-squared values. Hierarchical linear models with multiple predictors (Explicit Measures Model, Explicit Measures + P-IAT, Explicit Measures + P-IAT + IC), showing incremental validity of implicit measures (P-IAT, IC) for the TGC on top of all explicit self-report measures. Bold values indicate significant predictors. The final pseudo-variance explanation in the trust score with all explicit and implicit predictors combined amounts to 49% (*R*^*2*^_*m*_ = .490).

In the next step, we therefore checked whether implicit relationship-relevant measures might explain variance in the TGC trust score on top of the variance explained by all self-report measures together (see Table 2 for single predictor models of our two implicit measures). Indeed, we noted that implicit partner valence (P-IAT, *p* = .016, increase in explained variance of 9% up to 37%; see Table 3, Explicit Measures + P-IAT), and the implicit confidence in knowing the partner (IC, *p* = .002, increase in explained variance of 12% up to 49%, see Table 3, Explicit Measures + P-IAT + IC) explained additional unique variance when added step-by-step as predictors of the trust score. Higher trust scores were thus associated with more positive partner valence and a higher confidence in knowing the partner. Together, these results demonstrate incremental, convergent validity of both implicit relationship-related measures on top of self-reported validation criteria and one another in predicting the trust score. They also suggest that the TGC captures variance explained by various self-report relationship-related measures, as well as variance explained by implicit relationship-related measures.

## Discussion

Our study presents the newly developed TGC as a standardized, reliable and valid tool for the assessment of trust in romantic relationships. Internal consistency scores of the TGC trust score indicated acceptable reliability. Trust scores correlated positively between romantic partners, and the mean score was positive, indicating participants’ willingness to invest their own resources in their partner’s trustworthiness while being in a committed relationship. While considerable levels of mutual trust can be expected in intact romantic relationships (e.g., Rempel, Holmes and Zanna [5] report an average self-reported trust score towards the partner of 5.63 out of 7 points), the normal distribution of positive trust scores attests for considerable variance in our sample while leaving the opportunity to investigate low or negative trust levels in dysfunctional relationships in future studies. Pointing to the discriminant validity of our measure, the trust score was unrelated to unspecific interpersonal trust [20], demonstrating the TGC’s specificity for assessing partner-specific trust. Furthermore, it seems to be independent of impression management, suggesting that the decisions and costly investments in the TGC successfully prevent socially desirable response tendencies. Importantly, we found considerable positive associations between the trust score and self-reported trust in the partnership, satisfaction with the partnership, satisfaction with the partner, and felt closeness with the partner, providing strong support for the TGC’s convergent validity. Moreover, incremental variance in the trust score could be explained by implicit partner valence and implicit confidence in knowing the partner. Together, our results indicate that the TGC is an efficient measure of partner-specific trust in romantic relationships, capturing the variance of various self-report and implicit relationship-related measures.

By operationalizing trust via an individual’s willingness to invest their own financial resources in their romantic partner’s pro-relationship attitudes (collected by dichotomous responses to relationship-relevant items), the TGC combines features and advantages of measures relying on costly behavior, implicit tests, and self-reports. First, the TGC shares features with economic paradigms modeling real-life social behavior by means of decisions in interactive games which entail actual financial consequences for all interacting partners present simultaneously during the game [41]. Romantic partners also complete the TGC in an interactive social setting, responding simultaneously to relationship-relevant items, and making their costly decisions in relation to their partner’s responses. The TGC thereby uniquely accounts for the fact that real-life trust behavior often means accepting a certain amount of risk by investing one’s own resources with the anticipation of future advantages. Second, the TGC involves self-reports on relationship-relevant issues whose selection was informed by research emphasizing the behavior of romantic partners in trust-diagnostic situations (e.g., experiences concerning the partner’s faithfulness, repeated reassurance of positive feelings towards the partner, or personal sacrifices for the partner and partnership during relationship difficulties) as being essential to developing mutual trust [2,4,42]. Consequently, the TGC operationalizes partner-specific trust not solely by an abstract monetary investment, but by specific monetary investments in the partner indicating pro-relationship attitudes concerning specific relationship-relevant situations. Third, like in indirect tests, the true purpose of the measurement remains unclear to participants during the TGC, thus minimizing the risk of socially desirable responses and enabling attitudes to be captured indirectly that might be introspectively inaccessible [32,43].

With all these advantages at hand, what are the consequences for a romantic relationship if partners demonstrate high willingness to invest their own resources in their romantic partner’s pro-relationship attitudes? Expanding upon previous research that demonstrated that trust is a key foundation of stable and satisfactory romantic relationships [3,4], we find that participants who have confidence concerning their partner’s pro-relationship attitudes display high satisfaction with the partnership and their partner, feel close to their partner, implicitly evaluate their partner as more positive compared to an attractive alternative, and reveal stronger implicit confidence in knowing their partner well when estimating his or her responses. Based on these correlative findings, we speculate that on the one hand, trust provides the basis for an increase in positive explicit and implicit perceptions of the partner and partnership, and conversely, that these perceptions lead to increased trust towards the partner. Future experimental studies using the TGC could further investigate the causal direction of these associations, as well as influences of other relationship-related constructs that we did not consider in our study (e.g., attributions, commitment and pro-relationship behavior, love, perceived security, responsiveness, attachment styles). With regard to partner-satisfaction, we noted that high trust scores associated specifically with high values on the Togetherness and Communication subscale of the PFB [24], expressing satisfaction with the partner’s tendency to communicate about events, feelings, and needs. One could speculate that partners have built up trust by having communicated their trustworthiness concerning some of the TGC’s potentially relationship-threatening situations (e.g., “I would stay with you if you had to work in another country for a longer time”). This speculation is in line with research indicating that individuals who communicate greater trust often have partners who report deeper trust [4,44]. Another clue for the significance of a high trust score comes from its association with implicit relationship-related measures which contributed to large portions of explained variance in the trust score. Participants achieving high trust scores implicitly evaluated their partner more positive compared to an attractive alternative and demonstrated higher implicit confidence in knowing their partner. High partner-specific trust might increase these implicit positive evaluations of the partner which in turn can shield the partnership from potentially threatening situations [45,46]. Future research applying longitudinal designs could investigate the bidirectional relationship between trust and these evaluations.

Taking a novel and innovative approach to measure trust towards a romantic partner, what are its prospects and limitations? First, the TGC operationalizes trust as the willingness to invest in pro-relationship attitudes of the romantic partner. Whereas the present game paradigm differs from the classical trust game played in an completely anonymous setting, our operationalization allows for measuring trust among romantic partners who know each other well. Based on our items, a high trust score reflects confidence in the partner to provide love and security, be faithful, and prove his or her loyalty during relationship challenges. These items might be complemented by further aspects of pro-relationship attitudes in future studies (e.g., the partner’s continuous dependability). Assuming that actual social behavior emerges as a function of both individual trait and situational state characteristics, it is plausible that the trust score constitutes a combination of both elements, which is in line with major theoretical models [4,5]. With regard to state influences on the trust construct, future studies could also explore whether the current mood during the experiment influences decisions in the TGC. We should also mention that our findings refer to a sample of mainly young students being in a relationship for just a few years, and cannot be generalized to other populations. Note that items in the TGC can easily be adapted to investigate different aspects of trust in alternative experimental settings, or other samples (e.g., married or older couples).

In conclusion, our study demonstrates that the TGC is a reliable and valid measure of partner-specific trust behavior in romantic relationships offering many potential applications within and beyond romantic relationship research. By uniquely assessing the willingness to invest one’s own resources in the partner’s pro-relationship attitudes, the TGC can provide new insights into how trust behavior is generated, sustained, and compromised in romantic relationships [3,4], and how it interacts with relationship-relevant dispositions (e.g., attachment style [47]). It could help to diagnose deficits or asymmetries of trust between partners, monitor the effects of therapeutic interventions, or help to initiate constructive communication on relationship-relevant issues presented in the TGC items (for research on the importance of communication in romantic relationships, see [48–51]). One could further identify dysfunctional trust in relationships in case of one partner or both partners suffering from a mental disorder that hampers the development of interpersonal trust (e.g., separation anxiety; [52]). Moreover, the TGC enables researchers to investigate experimental manipulations of various social contexts (e.g., couple conflict; [53,54]) and trust-related hormones (e.g., oxytocin; [8]) on partner-specific trust. Beyond assessing trust in romantic relationships, the TGC could also be flexibly adapted to assess trust in alternative dyadic relationships such as those involving friends, family members, work colleagues, or members of distinct social groups. In sum, our study supports the TGC’s utility as a standardized tool for assessing trust behavior in dyadic relationships, which may stimulate future research in various contexts to improve our understanding of trust in close human social relationships.

## Supporting information

S1 Fig Instructions for the Trust Game for Couples (TGC). Instructions and comprehension questions for the TGC, introduced to participants as the “partner decision game”. “Correct” is the right answer to all comprehension questions a) to e). For f), the correct answer for Partner A is -10 cents and for partner B +57 cents.
(PDF)

S2 Fig Distributions of the nine separate trust scores. Separate trust scores of the nine trust-relevant rounds in the TGC each have a possible range from -6 to 6. Please note that each trust score results from investing in either pro-relationship or anti-relationship attitudes of the partner collected in stage 3. On average, participants spent 59.38% of their initial resources in the Trust Game for Couples (SD = 21.15%). Participants invested 88.49% (SD = 16.08%) of their spent resources in pro-relationship attitudes, and 11.51% (SD = 16.08%) in anti-relationship attitudes.
(TIF)

S3 Fig Stimuli of the P-IAT: Attribute dimension. Pictures with positive and negative valence in the Partner Implicit Association Test (P-IAT), taken from the International Affective Picture System [33].
(PDF)

S4 Fig Stimuli of the P-IAT: Target category partner. The target-category Partner in the Partner Implicit Association Test (P-IAT) consists of 4 individually chosen stimuli: The partner’s first name, the partner’s last name, a characteristic hobby and a typical character trait. The partner’s first and last name are typed in manually by participants; for the choice of a characteristic hobby and character trait the presented lists are offered.
(PDF)

S5 Fig Stimuli of the P-IAT: Target category attractive alternative. The target-category A in the Partner Implicit Association Test (P-IAT) consists of 4 stimuli, including first name, last name, characteristic hobby and character trait. These stimuli are presented to participants after their individual choice of the most attractive out of four opposite sex faces. Attractive faces were taken from the Chicago Face Database [34].
(PDF)

S1 Table The 15 items of the TGC and descriptive properties of separate trust scores. Codes T1-T9 = Trust-relevant items, Codes D1-D6 = Distractor-items. Items as shown are presented to participants at the beginning of each round (stage 1), and can be answered “Yes” or “No”. In stage 2, items are presented in the form of: “My partner is absolutely sure that he/she loves me” (T6). Descriptive properties (M, SD, Range) are presented on the separate trust scores of each relationship-relevant item with possible values of -6 (50 cents investment in an anti-relationship attitude of the partner) to 6 (50 cents investment in a pro-relationship attitude of the partner). Means of separate trust scores represent item difficulties, ranging from 1.31 (T1, most difficult item) to 5.08 (T6, easiest item). Note, that descriptive properties of distractor items don’t represent separate trust scores and are thus unrelated to the final trust score. See S1 Fig for distributions of separate trust scores.
(PDF)

S2 Table The seven blocks of the Partner Implicit Association Test (P-IAT). Block-structure of the P-IAT (Greenwald et al., 1998). Before practicing the categorization of the target-stimuli (Block 2), participants were instructed to memorize the assignment of the 8 stimuli for 30 seconds. The block sequence was randomized, so half of the subjects completed the paradigm as shown in the table, while the others started with incongruent blocks (switch 2, 3, 4 with 5, 6, 7).
(PDF)

S1 Dataset Excel-file containing the analyzed data set. (XLSX)
