# Supplementary material for: The Trust Game for Couples (TGC): A new standardized paradigm to assess trust in romantic relationships
Source: PLoS One. 2020 Mar 26;15(3):e0230776. doi: 10.1371/journal.pone.0230776 (PMC7098626; doi:10.1371/journal.pone.0230776)
Supplement: S1 Fig — Instructions and comprehension questions for the TGC, introduced to participants as the “partner decision game”. “Correct” is the right answer to all comprehension questions a) to e). For f), the correct answer for Partner A is -10 cents and for partner B +57 cents. (PDF) [file pone.0230776.s001.pdf]

Instructions for the partner decision game

Welcome to the partner decision game! In this game, it will be your task to estimate your partner's decisions as well as possible. Knowing your partner well will be rewarded! Together, you and your partner can earn a maximum double-digit extra amount to your shared profit!

In the game, statements will be presented that can be answered with "Yes", or "No". Please answer the questions honestly, which can be very personal. **All answers are anonymous, and will be visible to neither our study-assistants nor your partner at any time.** Next, you will estimate which honest answer you expect your partner will provide. The problem is: you are not allowed to communicate with each other! Do you still want to place a bet that you can estimate your partner's answer correctly?

- Rules -

- In 15 playing-rounds, 15 statements will be presented to you that have something to do with your relationship with your partner.
- Example: „When going to the movies together, I usually let my partner decide which film to watch."
- In each round, you'll make three decisions concerning the presented statement:

  Decision 1: Answer honestly by stating "Yes", or "No".

  Decision 2: Decide which answer you expect your partner will provide.

  Decision 3: Place a bet that you correctly estimated your partner's answer.

- Answer spontaneously, and don't think for too long! The next screen will only appear if you and your partner both clicked "Continue". This means, that you and your partner are playing all rounds of the game simultaneously.
- In each playing-round you'll be provided with a sum of 50 cents. You can place bets of 0, 10, 20, 30, 40 or 50 cents on estimating your partner's answers correctly.
- The more you bet, the more you can win! However, the profit margin drops the higher you bet. You should therefore only place high bets if you feel extremely confident about how your partner will answer. If your estimation is false, you lose your bet! Here you can see all the possible bets and resulting profits for one playing-round:

Possible bets:	Winning profit:	Lost profit:
**0 cent**	50 cents	50 cents
**10 cents**	57 cents	40 cents
**20 cents**	61 cents	30 cents
**30 cents**	63 cents	20 cents
**40 cents**	64 cents	10 cents
**50 cents**	65 cents	0 cent

- **Important:** Your decisions remain anonymous, meaning that neither your partner nor our study assistants are informed about any of your decisions at any point during the game.
- The individual profits of yourself and your partner are combined to create a shared profit at the end of the game, so neither you nor your partner will be informed as to who has earned a higher

profit. It is thus impossible to know or figure out how specific decisions were made from the profit!

- **Tip:** The goal of the game for you and your partner is to estimate each other as honestly as possible. There are no “true” or “false” answers, and all your decisions will remain anonymous. Please answer the statements spontaneously and honestly. In that way, your partner will find it easier to estimate how you will answer. For you, too, it will only be possible to estimate your partner’s decisions well if you can rely on him or her to give honest answers.

- Comprehension questions -

a) In this game I will try to estimate how my partner will answer certain statements.

Correct ◯ Incorrect ◯

b) My partner and I will play for a shared profit.

Correct ◯ Incorrect ◯

c) I won’t get any information on my partner’s answers in the game. My partner won‘t get information on my answers either.

Correct ◯ Incorrect ◯

d) The more confident I feel, the more money I should bet on estimating my partner’s answer correctly.

Correct ◯ Incorrect ◯

e) I should answer the questions honestly to maximize the profit I’ll share with my partner.

Correct ◯ Incorrect ◯

f) Partner A answers „Yes“. He believes that partner B will answer „No“, and bets 40 cents that that estimation is true. Partner B answers „Yes“. She believes that partner A will answer „Yes“, and places a 10-cent bet on her estimation being true. How much money can each player contribute to the shared profit in this playing-round?

Partner A: ______________________

Partner B: ______________________
