# Supplementary figures and images for: The Trust Game for Couples (TGC): A new standardized paradigm to assess trust in romantic relationships
Source: PLoS One. 2020 Mar 26;15(3):e0230776. doi: 10.1371/journal.pone.0230776 (PMC7098626; doi:10.1371/journal.pone.0230776)

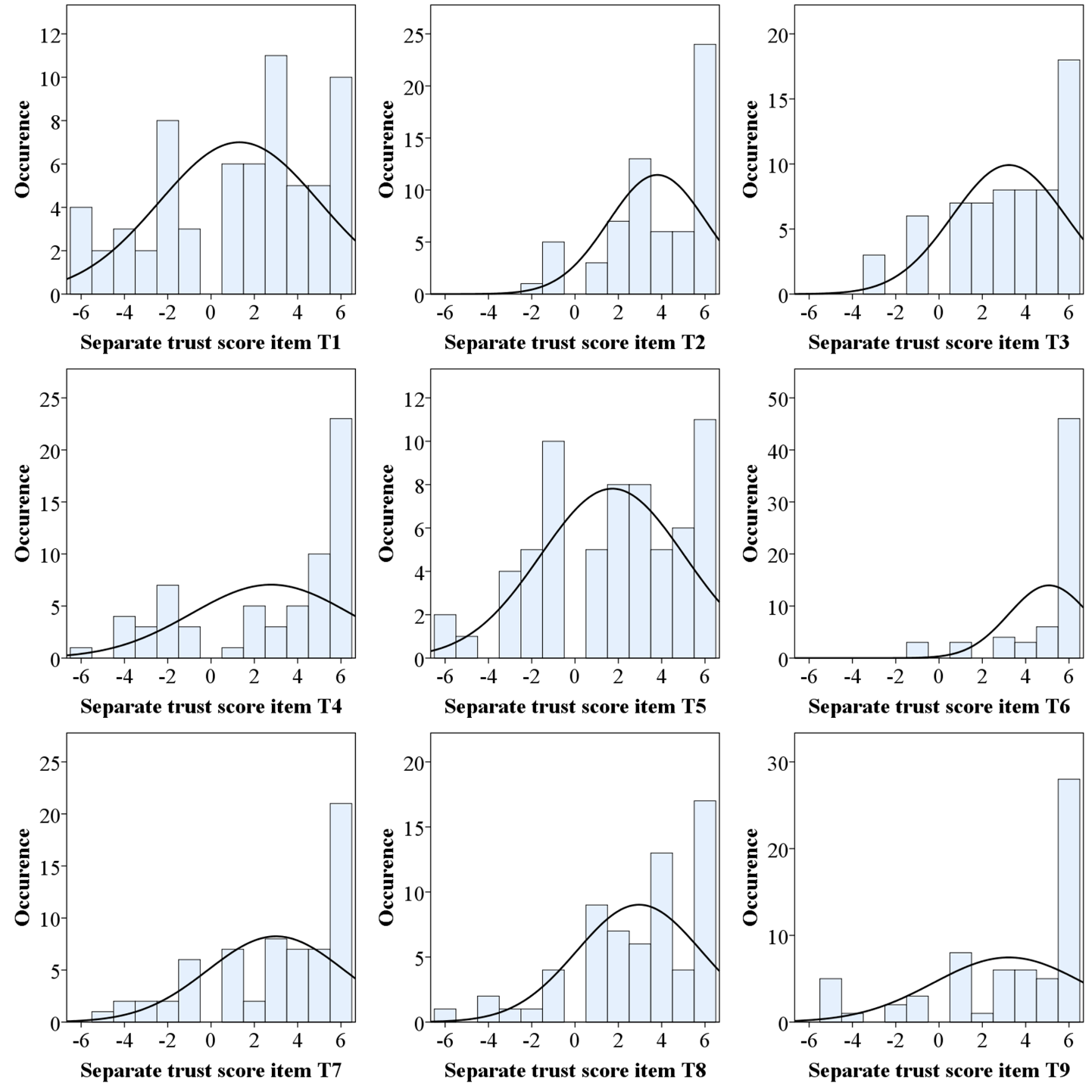

Supplement: S2 Fig — Separate trust scores of the nine trust-relevant rounds in the TGC each have a possible range from -6 to 6. Please note that each trust score results from investing in either pro-relationship or anti-relationship attitudes of the partner collected in stage 3. On average, participants spent 59.38% of their initial resources in the Trust Game for Couples (SD = 21.15%). Participants invested 88.49% (SD = 16.08%) of their spent resources in pro-relationship attitudes, and 11.51% (SD = 16.08%) in anti-relationship attitudes. (TIF) [file pone.0230776.s002.tif]

Attribute category: POSITIVE

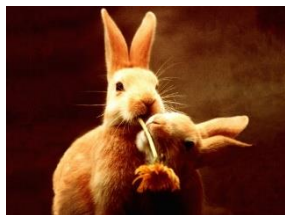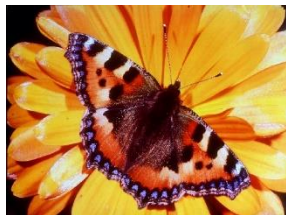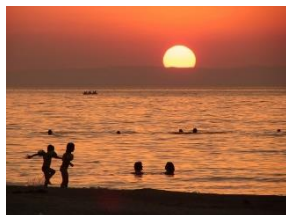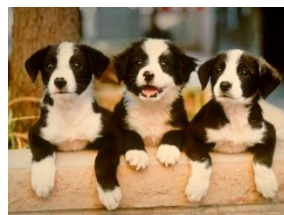

Attribute category: NEGATIVE

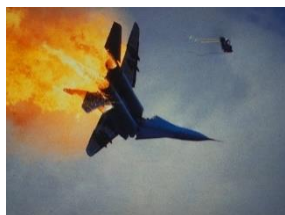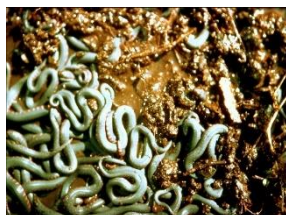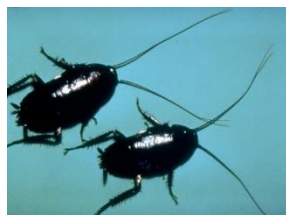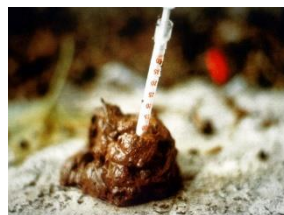

Supplement: S3 Fig — Pictures with positive and negative valence in the Partner Implicit Association Test (P-IAT), taken from the International Affective Picture System [33]. (PDF) [file pone.0230776.s003.pdf]
