# Supplementary material for: The Trust Game for Couples (TGC): A new standardized paradigm to assess trust in romantic relationships
Source: PLoS One. 2020 Mar 26;15(3):e0230776. doi: 10.1371/journal.pone.0230776 (PMC7098626; doi:10.1371/journal.pone.0230776)
Supplement: S4 Fig — The target-category Partner in the Partner Implicit Association Test (P-IAT) consists of 4 individually chosen stimuli: The partner’s first name, the partner’s last name, a characteristic hobby and a typical character trait. The partner’s first and last name are typed in manually by participants; for the choice of a characteristic hobby and character trait the presented lists are offered. (PDF) [file pone.0230776.s004.pdf]

Possible choices for the partner's characteristic hobby:

baking	badminton	ballett	band	basketball	handicraft work	bouldering
cello	choire	comics	computer-games	electric guitar	watch TV	fitness-center
flute	fotography	soccer	gardening	parlor games	guitar	crochet
hip hop	skating	jazz	jogging	keyboard	cinema	clarinet
piano	climbing	cooking	reading	painting	musicals	sewing
orchestra	opera	parties	pilates	bike	horseback riding	sauna
saxophone	drums	swimming	sailing	shopping	singing	ski
slackline	snowboard	squash	dancing	diving	tennis	theatre
table tennis	pottery	violin	volleyball	hiking	yoga	drawing

Possible choices for the partner's character trait:

balanced	enthusiastic	modest	disciplined	honest
ambitious	empathic	fair	imaginative	patient
calm	righteous	sociable	generous	helpful
polite	humorous	caring	courageous	indulgent
tidy	optimistic	punctual	respectful	quick-witted
sensitive	self-confident	animal-loving	tolerant	uncomplicated
ecologically sensitive	cosmopolitan	eager for knowledge	determined	reliable
