# Supplementary material for: The Trust Game for Couples (TGC): A new standardized paradigm to assess trust in romantic relationships
Source: PLoS One. 2020 Mar 26;15(3):e0230776. doi: 10.1371/journal.pone.0230776 (PMC7098626; doi:10.1371/journal.pone.0230776)
Supplement: S5 Fig — The target-category A in the Partner Implicit Association Test (P-IAT) consists of 4 stimuli, including first name, last name, characteristic hobby and character trait. These stimuli are presented to participants after their individual choice of the most attractive out of four opposite sex faces. Attractive faces were taken from the Chicago Face Database [34]. (PDF) [file pone.0230776.s005.pdf]

Pictorial choices for the ATTRACTIVE ALTERNATIVE for the female partner:

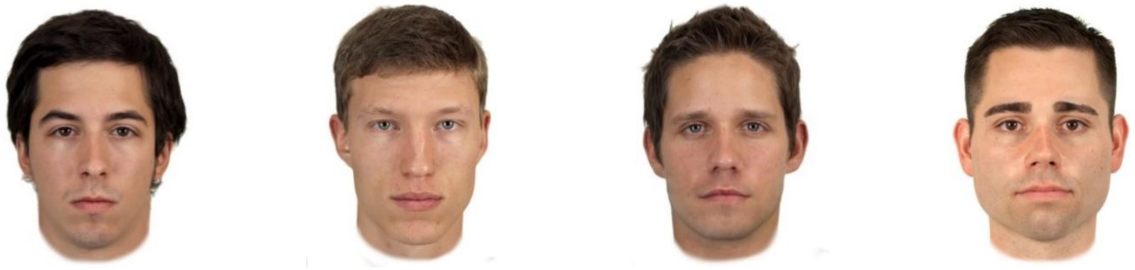

Pictorial choices for the ATTRACTIVE ALTERNATIVE for the male partner:

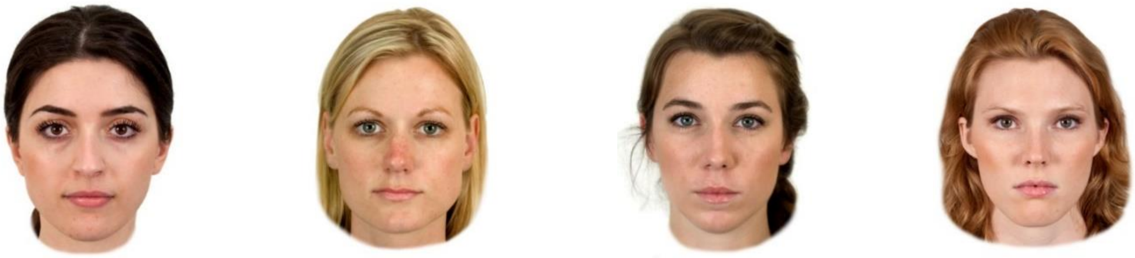

After choosing the face they are most attracted to, participants are shown information on this person as follows:

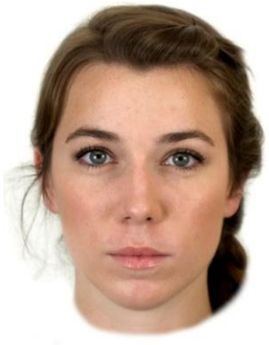

First name:	Antonia
Last name:	Bauer
Hobby:	Cello
Character trait:	Reliable
