# Supplementary material for: The Trust Game for Couples (TGC): A new standardized paradigm to assess trust in romantic relationships
Source: PLoS One. 2020 Mar 26;15(3):e0230776. doi: 10.1371/journal.pone.0230776 (PMC7098626; doi:10.1371/journal.pone.0230776)
Supplement: S1 Table — Codes T1-T9 = Trust-relevant items, Codes D1-D6 = Distractor-items. Items as shown are presented to participants at the beginning of each round (stage 1), and can be answered “Yes” or “No”. In stage 2, items are presented in the form of: “My partner is absolutely sure that he/she loves me” (T6). Descriptive properties (M, SD, Range) are presented on the separate trust scores of each relationship-relevant item with possible values of -6 (50 cents investment in an anti-relationship attitude of the partner) to 6 (50 cents investment in a pro-relationship attitude of the partner). Means of separate trust scores represent item difficulties, ranging from 1.31 (T1, most difficult item) to 5.08 (T6, easiest item). Note, that descriptive properties of distractor items don’t represent separate trust scores and are thus unrelated to the final trust score. See S1 Fig for distributions of separate trust scores. (PDF) [file pone.0230776.s006.pdf]

S1 Table. The 15 items of the TGC and descriptive properties of separate trust scores.

Code	Item (Statement)	M	SD	Range
T1	I am not sure I would want a long-distance relationship with my partner if he/she had to work in another country for a longer period. (inverse)	1.31	3.70	-6 – 6
T2	I would stick with my partner if he/she needed care after becoming ill or having an accident.	3.80	2.27	-2 – 6
T3	I might leave my partner if he/she only had time for me during weekends due to his job. (inverse)	3.23	2.62	-3 – 6
T4	I am sure that I want to grow old with my partner.	2.77	3.68	-6 – 6
T5	I am sometimes not entirely sure whether my partner is the right one for me. (inverse)	1.72	3.32	-6 – 6
T6	I am absolutely sure that I love my partner.	5.08	1.86	-1 – 6
T7	Since being together with my partner, I avoid getting too close to other attractive men/women.	3.00	3.15	-5 – 6
T8	I feel much less attached to other men/women since being with my partner.	2.95	2.88	-6 – 6
T9	I am absolutely sure I would never cheat on my partner.	3.22	3.48	-5 – 6
D1	I usually take advice from my partner when buying new clothes.	-	-	-
D2	I believe that I would beat my partner in a chess game.	-	-	-
D3	I would like to go on a long cruise with my partner.	-	-	-
D4	I like dining in expensive restaurants with my partner.	-	-	-
D5	I don’t know my partner’s shoe-size.	-	-	-
D6	I already have an idea what to give my partner for his/her next birthday.	-	-	-
