# Supplementary material for: The Trust Game for Couples (TGC): A new standardized paradigm to assess trust in romantic relationships
Source: PLoS One. 2020 Mar 26;15(3):e0230776. doi: 10.1371/journal.pone.0230776 (PMC7098626; doi:10.1371/journal.pone.0230776)
Supplement: S2 Table — Block-structure of the P-IAT (Greenwald et al., 1998). Before practicing the categorization of the target-stimuli (Block 2), participants were instructed to memorize the assignment of the 8 stimuli for 30 seconds. The block sequence was randomized, so half of the subjects completed the paradigm as shown in the table, while the others started with incongruent blocks (switch 2, 3, 4 with 5, 6, 7). (PDF) [file pone.0230776.s007.pdf]

**S2 Table. The seven blocks of the Partner Implicit Association Test (P-IAT).**

| **Block** | **Task** | **Button “L”** | **Button “R”** | **Items** |
|---|---|---|---|---|
| **1** | Practice Attribute | Negative | Positive | 16 (8 items x2) |
| **2** | Practice Target | Alternative | Partner | 16 (8 items x2) |
| **3** | Practice Congruent | Negative + Alternative | Positive + Partner | 32 (16 items x2) |
| **4** | Congruent Block | Negative + Alternative | Positive + Partner | 64 (16 items x4) |
| **5** | Practice Target switch | Partner | Alternative | 16 (8 items x2) |
| **6** | Practice Incongruent | Negative + Partner | Positive + Alternative | 32 (16 items x2) |
| **7** | Incongruent Block | Negative + Partner | Positive + Alternative | 64 (16 items x4) |
